# Transcriptome Patterns from Primary Cutaneous *Leishmania braziliensis* Infections Associate with Eventual Development of Mucosal Disease in Humans

**DOI:** 10.1371/journal.pntd.0001816

**Published:** 2012-09-13

**Authors:** Ana Claudia Maretti-Mira, Jaime Bittner, Manoel Paes Oliveira-Neto, Minghsun Liu, Dezhi Kang, Huiying Li, Claude Pirmez, Noah Craft

**Affiliations:** 1 Division of Molecular Medicine, Department of Medicine, Los Angeles Biomedical Research Institute at Harbor-UCLA Medical Center, Torrance, California, United States of America; 2 Laboratório Interdisciplinar de Pesquisas Médicas, Instituto Oswaldo Cruz, Fiocruz, Rio de Janeiro, Rio de Janeiro, Brazil; 3 Ambulatório de Leishmanioses, Instituto de Pesquisas Clínicas Evandro Chagas, Fiocruz, Rio de Janeiro, Rio de Janeiro, Brazil; 4 Infectious Diseases Section (111 F) and Research Service, VA Medical Center West Los Angeles, Los Angeles, California, United States of America; 5 David Geffen School of Medicine, University of California Los Angeles, Los Angeles, California, United States of America; 6 Department of Molecular and Medical Pharmacology, Crump Institute for Molecular Imaging, UCLA-DOE Institute for Genomics and Proteomics, Los Angeles, California, United States of America; Yale School of Public Health, United States of America

## Abstract

**Introduction:**

Localized Cutaneous Leishmaniasis (LCL) and Mucosal Leishmaniasis (ML) are two extreme clinical forms of American Tegumentary Leishmaniasis that usually begin as solitary primary cutaneous lesions. Host and parasite factors that influence the progression of LCL to ML are not completely understood. In this manuscript, we compare the gene expression profiles of primary cutaneous lesions from patients who eventually developed ML to those that did not.

**Methods:**

Using RNA-seq, we analyzed both the human and *Leishmania* transcriptomes in primary cutaneous lesions.

**Results:**

Limited number of reads mapping to *Leishmania* transcripts were obtained. For human transcripts, compared to ML patients, lesions from LCL patients displayed a general multi-polarization of the adaptive immune response and showed up-regulation of genes involved in chemoattraction of innate immune cells and in antigen presentation. We also identified a potential transcriptional signature in the primary lesions that may predict long-term disease outcome.

**Conclusions:**

We were able to simultaneously sequence both human and *Leishmania* mRNA transcripts in primary cutaneous leishmaniasis lesions. Our results suggest an intrinsic difference in the immune capacity of LCL and ML patients. The findings correlate the complete cure of *L. braziliensis* infection with a controlled inflammatory response and a balanced activation of innate and adaptive immunity.

## Author Summary

In Brazil, American tegumentary leishmaniasis is mainly caused by *Leishmania braziliensis* infection. Usually, it begins as a solitary skin lesion, which is called Localized Cutaneous Leishmaniasis (LCL). However, after this lesion heals, 5% of the patients may develop destructive lesions of the mucosa of nose and throat, which is called Mucosal Leishmaniasis (ML). Currently, there is no technology to identify individuals at risk for ML, and the factors that control the evolution to ML remain unknown. This work aims to study the human gene expression patterns that may contribute to the clinical manifestation of the disease. We used the RNA-Seq technique to study skin lesions from individuals that had LCL (LCL group) and those who developed ML (ML group). Our results suggest that individuals that progressed to ML expressed low levels of genes involved in the immune and inflammatory responses, which might lead to insufficient control of the infection. We were also able to identify a potential gene expression signature to predict long-term disease outcome.

## Introduction

Leishmaniasis is an important parasitic disease found in 88 tropical and subtropical countries and is still one of the most neglected diseases in the world. Approximately 12 million people are infected with the *Leishmania* protozoa, 270 thousand new cases occur yearly, and 1.6 million individuals are considered at risk of infection. American tegumentary leishmaniasis (ATL) is a serious public health problem in Latin America. In Brazil, over 26 thousand new cases are registered annually and most of the infections are caused by *Leishmania braziliensis* [1], [2].

Localized cutaneous leishmaniasis (LCL) is the most common manifestation of ATL and is characterized by the development of a single lesion at the site of the sandfly's bite that tends to heal spontaneously and gradually even if left untreated [3]. However, the parasite dissemination from the primary cutaneous lesion to distant skin and mucosal regions, without lesion development, is a common event. *L. braziliensis* infection can still be detected sometimes after clinical cure [4]. Fortunately, less than 5% of the individuals infected with *L. braziliensis* may progress to mucosal leishmaniasis (ML), which results in ulceration of oropharynx and nasal mucosa either at the time of primary skin lesion or several months or years later [5]. The natural history of ML consists in the progressive destruction of the nasal septa and soft and hard palates, eventually causing severe facial disfiguration and respiratory disturbances that do not heal spontaneously [6]. Moreover, 22–50% of the individuals with ML are unresponsive to the current antimonial therapy and have an associated increase in morbidity and mortality [7].

Susceptibility to ML progression has been associated with polymorphism of *TNF* (−308 bp), *IL6* (−174G/C) and *CCL2/MCP1* (−2518A/G) genes [8]–[10]. This, together with the finding of an increased frequency of HLA-DQw3 allele in ML patients, suggests that host genetic factors may play an important role in individual tendency to ML development [11]. However, these findings alone cannot explain susceptibility patterns entirely.

Following the genome sequencing of humans and *L. braziliensis*, and the development of Next Generation Sequencing (NGS) technologies [12], [13], we employed the RNA-seq method to analyze the transcriptomes of human and *L. braziliensis* in a high-throughput manner. The resulting data reveal both the identity and quantity of the different transcripts present from both host and pathogen simultaneously in infected tissue samples.

A better understanding of the immune environment of the primary infection site may allow us to identify the mechanisms involved in successful host defense, predict the factors involved in the disease outcome and potential pathways for targeted therapeutic intervention. We hypothesized that the quality of the host immune response at the primary cutaneous infection with *L. braziliensis* is responsible for the ultimate clinical outcome of the disease (cure or development of ML) and can be decoded by specific transcriptional profiles. To test this hypothesis, we characterized and compared the transcriptomes of the primary cutaneous lesions from both LCL and ML patients (before they developed ML). In addition to human transcripts, we were able to detect the *L. braziliensis* transcripts *in situ*, as well.

## Methods

An extended methodology section is available in the Supporting Materials and Methods on line (Text S1).

### Sample origin

Skin tissue fragments were obtained from biopsies of active primary cutaneous leishmaniasis lesions from 10 patients before starting treatment with pentavalent antimony. The biopsy procedure was performed under anesthesia and in sterile conditions. The fragments were collected from the border of the ulcer, including epidermis and dermis, immediately embedded in O.C.T. medium (Tissue-Tek, Sakura Finetek, USA) and preserved in liquid nitrogen. All patients lived in Rio de Janeiro state, Brazil, did not present any symptom of mucosal lesions and no other pathologies. After diagnose confirmation for infection with *L. braziliensis*, those patients were treated with Glucantime (Rhodia Laboratories, Antony, France). All patients completely resolved the primary cutaneous lesions after treatment, with no lesion relapse or need for repeat treatment. Five individuals cured completely after therapy (LCL group) and 5 individuals developed mucosal lesions long after initial healing of the primary lesion (ML group). The age of the patients ranged between 15–67 years in LCL group and 23–63 years in ML group. All five LCL individuals and 3 ML subjects developed only one primary cutaneous lesion. Two ML subjects had more than one cutaneous lesion. By patient recall, the average (± standard deviation) time of lesion evolution was similar in both groups (LCL: 1.4±0.5 months; ML: 2.3±1.3 months). The criteria for inclusion in this study, therefore, were the time of evolution of the lesion, which should range between 1–4 months; the good response for the treatment, which comprehended the completely re-epithelialization of the cutaneous lesion and no lesion reactivation; and the final outcome of the disease, which should be no disease relapse (LCL) or development of lesions at the nasopharynx mucosa (ML).

### Ethical issues

The research was approved by the Research Ethics Committee and IRB (Institutional Review Board) from both FIOCRUZ and LABIOMED Institutes. Written informed consent was obtained from all the adults and from the parents or legal guardians on behalf of the participants under 18 years of age. The study was conducted in agreement with the principles of the Helsinki Declaration and the Resolution 196/96 of the National Health Council of the Ministry of Health that regulates research involving human subjects in Brazil.

### Transcriptome library construction

The total RNA was isolated using guanidine-chloroform-phenol nucleic acid extraction, treated with DNAse (Qiagen, Valencia, USA) and cleaned with Rneasy Micro-kit (Qiagen). The quality control step was performed using Bioanalyzer (Agilent Technologies, Santa Clara, USA). Afterward, the total RNA was used to prepare the transcriptome libraries with the mRNA-seq sample preparation kit (Illumina, San Diego, USA) according to the manufacturer's instructions and sequenced using the Illumina Genome Analyzer II platform. The sequence of all cDNA libraries will be freely available on the GEO NCBI database (http://www.ncbi.nlm.nih.gov/geo), accession number GSE33601.

### Bioinformatic and biostatistic analyses

Reads were aligned to the references genomes using the Bowtie aligner and Tophat program. The reference genomes used in this project were from *Homo sapiens* (UCSC- hg19), *Leishmania braziliensis* (TritrypDB, version 3.2), *Staphylococcus aureus* (Wellcome Trust Sanger Institute - EMRSA15) and *Pseudomonas aeruginosa* (Wellcome Trust Sanger Institute - LEB58) (Table S8). SAMtools were used for quality control and processing the reads (Table S9). Cuffdiff from the Cufflinks' package was applied to normalize the number of human reads to FPKM (Fragments Per Kilobase per Million) considering gene isoforms and including multi-hit reads (up to 30 regions - Table S2). *Student's t-test* was used for dimensional reduction of the data set, considering as threshold *P*-values lower than 0.05 and the software SAM (Significance Analysis of Microarray [14]) was selected to perform the multiple testing correction, considering *q*-values lower than 0.01. Afterward, the genes with a minimum fold change of gene expression of 1.5 were considered for biological interpretation. Linear regression analysis was used to evaluate the similarity of gene expression between LCL and ML samples. A MA-plot was used to visualize the gene expression distribution in both groups. A rarefaction curve was calculated for each sample (method described in Text S1) to evaluate if the sequencing depth has provided a sufficient coverage of the transcriptome. Leave-One-Out cross validation analysis was performed as previously described [15], [16] to determine the phenotypic predictive power of the top differentially expressed genes. Functional annotation and canonical pathway identification of the selected genes was performed by Ingenuity Pathway Analysis (IPA) software (Ingenuity System, Redwood City, CA, http://www.ingenuity.com), and by Database for Annotation, Visualization and Integrated Discovery (DAVID) Bioinformatics Resources 6.7 (NIAID-NIH, http://david.abcc.ncifcrf.gov/home.jsp). The Euclidean distance method was used for hierarchical clustering of the samples using Gene Cluster 3.0 software (http://rana.lbl.gov/EisenSoftware.htm).

## Results

### RNA-seq technique effectively distinguished transcripts from two different species simultaneously

To determine the relative abundance of mRNA of individual genes for both host and pathogen simultaneously and in an unbiased manner, we used the RNA-Seq technique. The samples used for this purpose were skin biopsy fragments obtained from the border of primary cutaneous lesions of patients infected with *L. braziliensis* from Rio de Janeiro, Brazil. The sequencing generated 13±5 million reads per sample. The reads were aligned to both human (hg19) and *L. braziliensis* (Tritryp, vs 3.2) genomes using the Bowtie and Tophat algorithms. This annotation of the human genome has 23,136 genes, while the *L. braziliensis (Lbr)* genome has 8,504 genes. The results indicated a good efficiency of the RNA-seq assay to identify transcripts from both host and parasite genomes in the cutaneous lesions of human leishmaniasis.

On average, 20% of the reads were not assigned to human or *L. braziliensis* genomes. Almost 80% of the total number of reads aligned to the human genome, while less than 1% of the total reads (range 0.06–0.91%) were aligned to *L. braziliensis*. The ratios of misalignment between human and parasite genomes ranged from 0.002% to 0.01% (Table S1). As the absolute number of *L. braziliensis* reads detected was insufficient to effectively cover the *L. braziliensis* transcriptome, we did not perform differential gene expression analysis on the parasite transcripts.

Bacterial RNA would not be predicted to be detected due to lack of polyadenylation. To verify that bacterial RNA was not present in the samples to a high degree, we aligned the reads to *Staphylococcus aureus* (EMRSA15) and *Pseudomonas aeruginosa* (LEB58) genomes, both representing the common bacteria causing secondary infection in cutaneous leishmaniasis lesions in Brazil [17]. Less than 0.005% of the unaligned reads matched to these potential contaminants.

### Host gene expression

To estimate if the RNA-Seq sequencing depth provided a satisfactory coverage of expressed human genes, we calculated rarefaction curves using reads that were mapped to unique regions of the human genome. The curves indicate that we have achieved good coverage in all samples. Regardless the total number of reads, in all samples we were able to detect a similar number of expressed genes (Figure S1).

**Figure 1:**
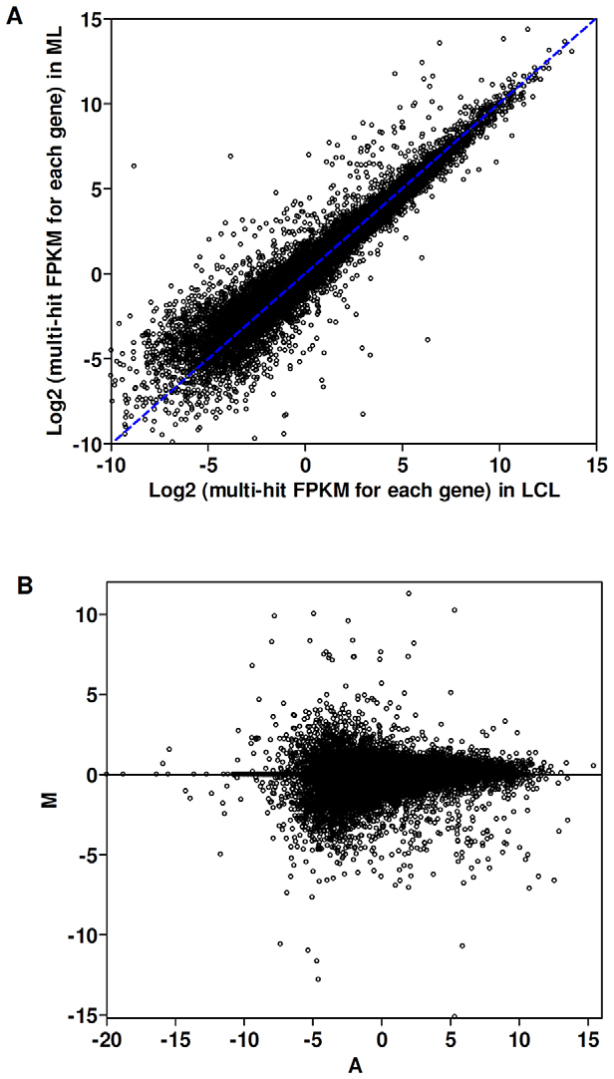
Similarities of gene expression patterns in LCL and ML samples. The analysis included reads that match to 1 to 30 regions of the human genome. The values were normalized to FPKM using the program Cuffdiff, which considers gene isoforms. A) The scatterplot compares the abundance of all human protein-coding genes detected by RNA-seq in LCL versus ML groups. Using linear regression analysis (blue dotted line), around 10% of the genes displayed divergence in the expression patterns (r^2^ = 0.91). B) The MA-plot shows the distributions of the gene expression between LCL and ML groups. The axis A indicates the intensity averages and the axis M indicates the intensity ratios. Dots located above zero in the M axis correspond to genes up-regulated in LCL group and dots located bellow zero in the same axis correspond to genes up-regulated in ML group. FPKM: Fragments Per Kilobase per Million. LCL: Localized Cutaneous Leishmaniasis. ML: Mucosal Leishmaniasis.

**Figure 2:**
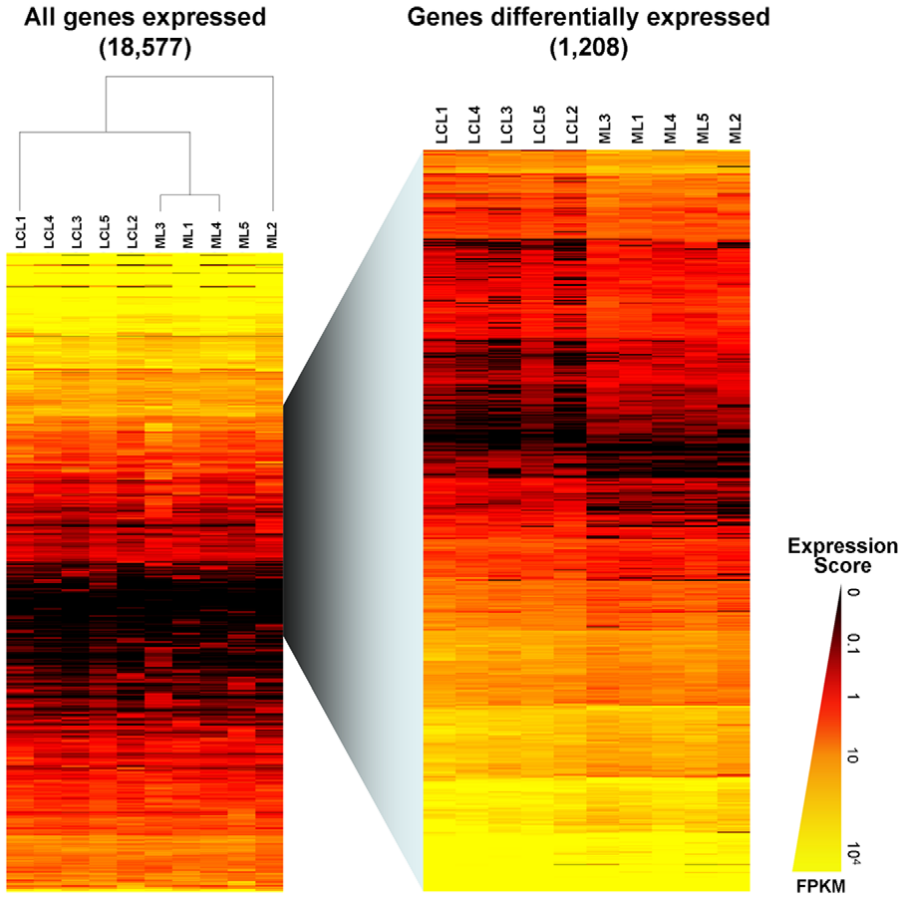
Global transcriptional expression patterns. Hierarchical clustering was performed using the Euclidean distance method (Gene Cluster 3.0) with complete linkage for both samples and genes assays. Intensity of gene expression increases from black to yellow. The heat map in the left panel includes the 18,577 genes detected by RNA-seq and shows that the samples were correctly clustered into the two clinical phenotypic groups, ML and LCL. The right panel represents the same assay using the 1208 genes selected by the dimensional reduction step performed by the *t-test*, confirming that the samples could still be clustered into LCL and ML groups. LCL: Localized Cutaneous Leishmaniasis. ML: Mucosal Leishmaniasis.

For gene expression analysis, unique and multi-hit reads that matched up to 30 regions to the human genome were considered and normalized to FPKM. The average percentage of reads that mapped in more than 2 regions was 23% (Table S2). Reads that aligned to more than 30 regions were excluded. The similarity of gene expression patterns of LCL and ML groups was analyzed by linear regression, which indicates that approximately 90% of the genes had comparable levels of expression (r^2^ = 0.91; Figure 1A). This similarity can also be observed in the MA-plot (Figure 1B). Additionally, using all genes that were expressed in at least one sample (18,577 genes), we performed hierarchical clustering on the samples using Euclidean distance (Figure 2 left panel). The heat map shows that all of the samples were correctly clustered into the two clinical phenotypic groups, ML and LCL.

Before determining the differences in the genes expression and in the biological pathways between the LCL and ML groups, we performed a dimensional reduction of the data set using *Student's t-test*, considering as threshold *P*-values lower than 0.05. This step resulted in the selection of 1208 genes (Figure 2 right panel). From this gene list, 58.5% (707) of the genes had increased expression in the LCL samples, while 41.5% (501) were increased in ML samples. Our choice of multiple testing correction was the software SAM (Significance Analysis of Microarray) which includes a special statistic called d statistic (true null is centered in 0) and includes randomization and permutation. We performed the multiple testing correction using the FPKM values. Considering the resulting *q*-value lower than 0.01 for significance, 1130 genes were selected. As an additional filter, we established a minimum fold change in the expression of 1.5. This step resulted in the selection of 867 genes (434 genes up-regulated in LCL group and 433 genes up-regulated in ML group). Those genes were considered as differentially expressed and potentially biologically relevant, and were then used for further biological interpretation.

### Gene classification according to biological function

We hypothesized that the genes that were differentially expressed between the ML (*n* = 5) and LCL (*n* = 5) clinical subgroups would represent important biological differences and would thus associate with different functional categories. We used Ingenuity Pathway Analysis (IPA) to determine the functional annotation of the differentially expressed genes (Table S3). As hypothesized, we observed several common biological activity pathways in both ML and LCL comprised of genes with distinct functions (Tables S4, S5, S6, S7).

**Figure 3:**
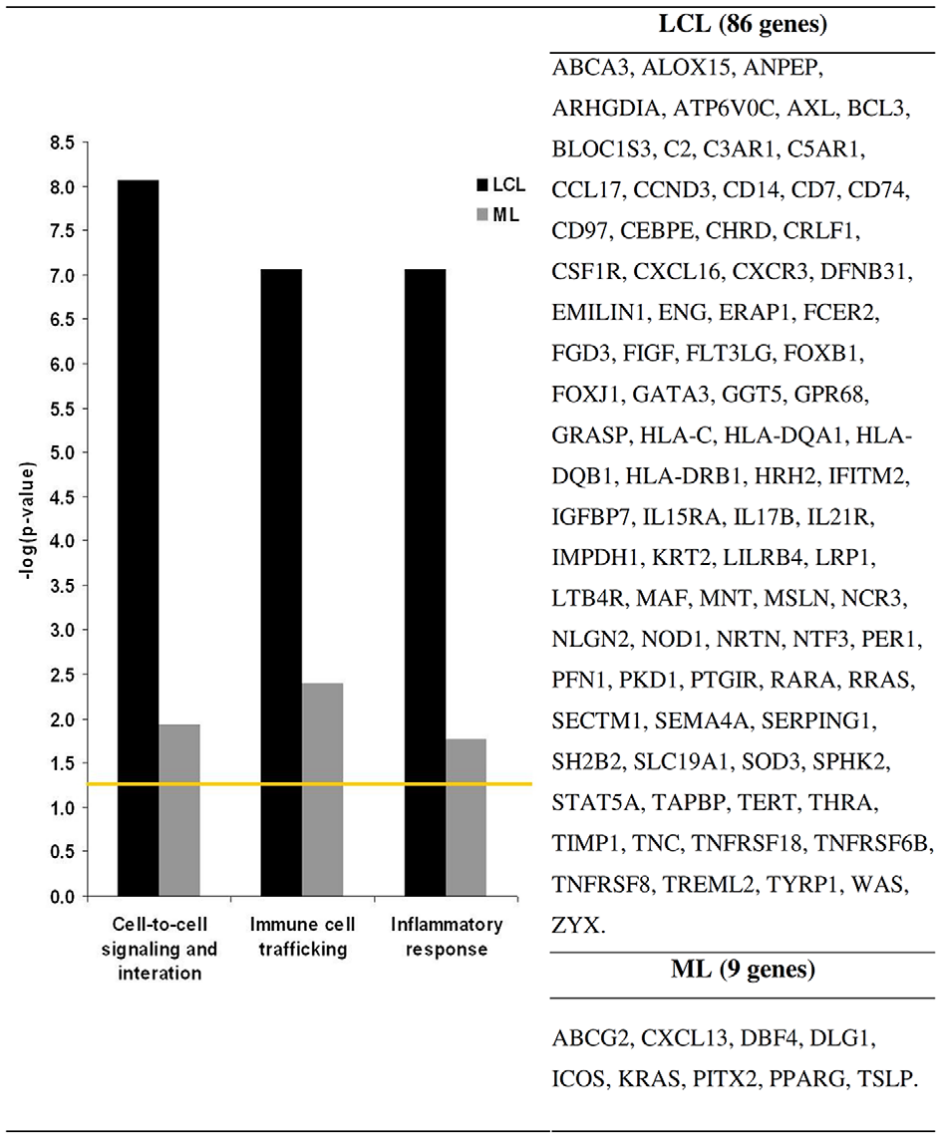
Genes involved in the most relevant biological activities according to Ingenuity Pathway Assay. The bars indicate the significance levels of the up-regulated genes presence based on the normal event (yellow line). Black and gray bars represent LCL and ML samples, respectively. LCL: Localized Cutaneous Leishmaniasis. ML: Mucosal Leishmaniasis.

The most significant finding was that many of the genes with increased expression in LCL lesions were involved in immune system activities, such as cell-to-cell signaling and interaction, immune cell trafficking, and inflammatory response. These groupings shared common events and involved 86 genes up-regulated in LCL samples (Tables 1 and 2, Figure 3). Immune response associated genes were related to the recruitment and activation of lymphocytes, granulocytes, natural killer cells and antigen presenting cells. In addition, we detected higher expression of genes related to the control of inflammation and induction of immune tolerance. Conversely, ML samples had only 9 genes with increased expression that were related to inflammation and immunity; all of them were related to recruitment of memory T cells and B lymphocytes (Table 2). The presence and implications of the most important individual genes and pathways is explored in more details in the discussion.

**Table 1:** Top 10 biological pathways observed in American tegumentary leishmaniasis lesions based on clinical presentation.

Localized cutaneous leishmaniasis	*P*-value ranges	# Genes
Cell-To-Cell Signaling and Interaction	8.49E-09–1.85E-02	75
Immune Cell Trafficking	8.75E-08–1.85E-02	47
Inflammatory Response	8.75E-08–1.92E-02	59
Infectious Disease	1.07E-06–1.85E-02	62
Cellular Growth and Proliferation	1.09E-06–1.85E-02	89
Dermatological Diseases and Conditions	8.35E-06–1.99E-02	62
Immunological Disease	1.95E-05–1.85E-02	80
Cellular Function and Maintenance	2.69E-05–1.85E-02	39
Inflammatory Disease	7.93E-05–1.99E-02	95
Cancer	1.63E-04–1.85E-02	101

Mucosal leishmaniasis	*P*-value ranges	# Genes
Cancer	2.80E-04–4.95E-02	80
Genetic Disorder	2.80E-04–4.88E-02	164
Metabolic Disease	2.80E-04–4.95E-02	73
Cell Death	8.31E-04–4.86E-02	37
Inflammatory Disease	8.64E-04–4.88E-02	12
Cellular Assembly and Organization	2.22E-03–4.95E-02	23
Gene Expression	2.71E-03–4.95E-02	10
Cell Cycle	2.79E-03–4.77E-02	37
Cell Morphology	2.98E-03–3.52E-02	30
Connective Tissue Development and Function	3.05E-03–4.95E-02	18

Assay using Ingenuity Pathway Analysis for gene classification according to functional annotation. Biological activities were ordered according to *P*-values. *# Genes* indicates the number of genes from each list that were involved in the biological activity.

**Table 2:** Upregulated events related to cell-to-cell signaling and interaction, immune cell trafficking and inflammatory response.

Localized Cutaneous Leishmaniasis		
Functional Categories	*P*-values	# Genes
Activation of leukocytes	8.75E-08	32
Immune response	4.71E-07	55
Recruitment of cells	4.55E-06	17
Activation of mononuclear leukocytes	4.24E-05	21
Recruitment of leukocytes	5.69E-05	14
Activation of lymphocytes	6.27E-05	20
Activation of T lymphocytes	4.74E-04	15
Recruitment of granulocytes	8.73E-04	9
Activation of antigen presenting cells	1.38E-03	11
T cell response	1.40E-03	7
Recruitment of lymphocytes	1.85E-03	6
Inflammatory response	3.41E-03	22
TH1 immune response	3.43E-03	5
Immune response of cytotoxic T cells	4.87E-03	2
Response of Th1 cells	6.52E-03	3
Cell movement of leukocytes	6.71E-03	20
Accumulation of dendritic cells	6.74E-03	2
Emigration of dendritic cells	8.88E-03	2
Response of lymphatic system cells	1.02E-02	6
Response of leukocytes	1.17E-02	10
Attraction of antigen presenting cells	1.32E-02	3
Recruitment of neutrophils	1.39E-02	6
Accumulation of leukocytes	1.43E-02	9
Attraction of cells	1.63E-02	5
Adhesion of cells	1.64E-02	25
Contact growth inhibition of eukaryotic cells	1.78E-02	6
Stimulation of leukocytes	1.78E-02	6
Antibody-dependent cell-mediated cytotoxicity	1.92E-02	3

Mucosal Leishmaniasis		
Functional Categories	*P*-values	# Genes
Migration of memory T lymphocytes	4.02E-003	2
Cell-cell adhesion of granulocytes	1.68E-002	1
Chemotaxis of B-1 lymphocytes	1.68E-002	1
Influx of B lymphocytes	1.68E-002	1
Migration of B-1 lymphocytes	1.68E-002	1
Recruitment of lymphoid cells	3.33E-002	1
Relocalization of B lymphocytes	3.33E-002	1
Formation of adherents junctions	4.77E-002	2
Adhesion of memory T lymphocytes	4.95E-002	1

Ingenuity Pathway Analysis. The most important functional categories participating in the three major biological activities selected for localized cutaneous leishmaniasis (LCL, *n* = 5) samples are listed above for both LCL and mucosal leishmaniasis (ML, *n* = 5) groups. Only events with *P*-values higher than 0.001 were included in this list. *# Genes* indicates the number of genes from each list that were involved in the events.

The majority of genes with increased expression in ML samples are involved in biological activities that would not have been intuitively predicted for this infectious disease. These biological groupings included cancer, genetic disorders, metabolic diseases, and cell death. Some of these activities were also observed in the LCL group, however, with less significance according to the IPA assay (Table 1, Table S3).

### Gene expression signature and biomarkers to predict clinical outcome

We hypothesized that transcriptional differences in the primary infectious lesions of cutaneous leishmaniasis could be used to predict the long term clinical outcome of the infection. To determine if the top differentially expressed genes could be used to predict the clinical form (ML *vs* LCL), we performed a Leave-One-Out cross validation analysis on the samples [15], [16]. Based on the groups of 5 ML subjects and 5 LCL subjects, the prediction algorithm determined that the top 13 differentially expressed genes showed the best prediction of the clinical phenotype (*P* = 0.12; Figure S2). According to the prediction, increased expression of the genes FBXL2, ZDBF2, MORN2, C1orf109, TSPAN13, MRPS31 and STAMBP, and decreased expression of CYB5R3, CREB5, EPB49, STMN3, ADCK1 and TMEM239 in a primary cutaneous lesion would indicate a higher probability of ML development (Table 3). Partly due to the small number of samples, the result was not statistically significant. However, this type of analysis could be effective when using a larger number of clinical samples.

**Table 3:** Genes associated with high risk for mucosal leishmaniasis progression.

Symbol	Entrez Gene Name	*P*-values	FC (Log2)
**Genes used in Leave-One-Out cross validation**			
ADCK1	aarF domain containing kinase 1	0.0013	−0.91
STMN3	Stathmin-like 3	0.0002	−0.86
EPB49	Erythrocyte membrane protein band 4.9 (dematin)	0.0008	−0.83
CREB5	cAMP responsive element binding protein 5	0.0012	−0.76
CYB5R3	Cytochrome b5 reductase 3	0.0004	−0.71
STAMBP	STAM binding protein	0.0003	0.75
MRPS31	Mitochondrial ribosomal protein S31	0.0013	0.98
TSPAN13	Tetraspanin 13	0.0012	0.99
C1orf109	Chromosome 1 open reading frame 109	0.0007	2.16
MORN2	MORN repeat containing 2	0.0002	2.36
ZDBF2	Zinc finger, DBF-type containing 2	0.0006	2.49
FBXL2	F-box and leucine-rich repeat protein 2	0.0006	2.71
TMEM239	Transmembrane protein 239	0.0008	NE
**Genes selected by IPA biomarker filter**			
TERT	Telomerase reverse transcriptase	0.0470	−3.32
NTF3	Neurotrophin 3	0.0342	−3.10
VWA2	Von Willebrand factor A domain containing 2	0.0274	−2.21
TNFRSF6B	Tumor necrosis factor receptor superfamily, member 6b, decoy	0.0442	−2.03
ALOX15	Arachidonate 15-lipoxygenase	0.0404	−1.74
FCER2	Fc fragment of IgE, low affinity II, receptor for (CD23)	0.0065	−1.68
SOX9	SRY (sex determining region Y)-box 9	0.0267	1.52
BRCA2	Breast cancer 2, early onset	0.0410	1.54
ARG2	Arginase, type II	0.0101	1.56
EYA2	Eyes absent homolog 2 (Drosophila)	0.0346	1.79
PPARG	Peroxisome proliferator-activated receptor gamma	0.0257	2.07
KLK4	Kallikrein-related peptidase 4	0.0432	3.82
AGR2	Anterior gradient homolog 2	0.0439	4.00
ABCG2	ATP-binding cassette, sub-family G (WHITE), member 2	0.0160	4.09
FGF1	Fibroblast growth factor 1 (acidic)	0.0131	4.57

Genes used in Leave-One-Out cross validation analysis or selected by Ingenuity Pathway Analysis biomarker filter tools. Possible biomarkers to predict the development of mucosal leishmaniasis were determined by IPA analysis and have a significant fold change and a predicted importance due to their use as diagnostic and prognostic biomarkers in other diseases.

FC: fold change. NE: No expressed.

Additionally, Ingenuity Pathway Analysis offers a biomarker filter tool based on a database of genes with known biological importance for diagnosis and prognosis for different diseases. Considering both FPKM values and the fold change for biomarker filtering, the IPA assay indicated 15 genes as possible markers of high risk to develop ML (Table 3). Based on the IPA analysis, patients that are likely to develop ML would demonstrate increased expression of the genes SOX9, BRCA2, ARG2, EYA2, PPARG, KLK4, AGR2, ABCG2 and FGF1 and decreased expression of TERT, NTF3, VWA2, TNFRSF6B, ALOX15 and FCER2.

## Discussion

Although several studies have addressed the immunological differences between LCL and ML patients, most of the observations were performed *in situ* using mucosal lesions, or systemically using PBMCs from patients with the active disease. The innovation brought by the present work is the comparison of LCL and ML groups starting from the primary cutaneous lesion, which is the beginning of both clinical forms. For this analysis, we used the RNA-seq technology to obtain and evaluate the transcriptome of those lesions. After Glucantime therapy, all patients successfully resolved the initial cutaneous lesions. Despite similar clinical control of the infection, we were able to detect consistent differences in the expression of genes involved in inflammatory and immune responses before the treatment, which could explain the persistence of the infection and ultimately predict the clinical outcome. These findings need to be validated in additional studies with more primary lesions in new patients.

Previous studies cannot be directly compared to this analysis mostly because of the different specimens used. However, for discussion, they are reviewed here. Our group had previously shown that mucosal lesions resulting from *L. braziliensis* infection displayed high levels of transcripts for IL-5, IL-10 and, specially, IL-4 when compared to cutaneous lesions [18]. Other authors observed the presence of increased number of cells producing IL-10 and IFN-γ in these type of lesions [19]. Nevertheless, the systemic approaches revealed that ML patients display higher levels of inflammatory cytokines, such as TNF-α and IFN-γ, and lower IL-10 production as compared with CL patients, besides a weak modulation by IL-10 and TGF-β [20]–[23]. Those studies also concluded that mucosal leishmaniasis is rather an immunopathologic hyperimmune reaction to parasites and their antigens [24].

Our findings revealed that several genes involved in diverse immunological activities, ranging from the innate immune response to adaptive immunity were expressed at lower levels in the primary cutaneous lesion from patients that afterward evolved to ML when compared to those who only presented LCL. This data set suggests that the quality of the immune response against *L. braziliensis* infection that best correlates with long term control of the disease involves the recruitment and activation of lymphocytes, dendritic cells (DC), macrophages, neutrophils, and natural killer (NK) cells. The specific transcripts detected do not guarantee the presence of specific cells or a predicted role for them. However, the high level of transcripts in LCL samples for genes involved in antigen presentation to both CD4^+^ and CD8^+^ T cells suggests this may be an important step for the development of a durable adaptive immune response against *Leishmania*. The genes TREM-2 and WAS have been associated with the migration of DCs to lymph nodes for antigen presentation [25], [26]. The detection of HLA-DRB1, HLA-DQA1, HLA-DQB1 and CD74 transcripts suggests a significant presence of professional antigen presenting cells (APC) *in situ*. The participation of HLA has been explored before in the immunopathology of leishmaniasis. The high frequency of some HLA-DR alleles was correlated to the susceptibility to mucosal leishmaniasis [11], while the levels of HLA-DR in cutaneous lesions were down-modulated after drug therapy [27]. Additionally, antigen presentation through MHC class I is supported by the high mRNA levels of endoplasmic reticulum aminopeptidase 1 (ERAP1), Tapasin (TAPBP) and HLA-C [28]–[30].

The transcriptome in LCL primary lesions also indicates a multi-polarized adaptive immune response. Expression of different chemokines, cytokines and receptors, transcription factor and others genes, suggests that the lymphocytes present in the lesions are Th1, Th2 and Th17, CD8^+^ cells, memory cells CD4^+^ and CD8^+^, natural killer T (NKT) cells. Some of these genes had been previously associated with leishmaniasis. IL-17 is detected in high levels in subclinical samples as compared to CL samples, suggesting the participation of Th17 in the control of the infection [31]. IL-21R, CD30 (TNRFSF8) and WAS participate of the Th2 polarization during *Leishmania* infection [32]–[34]. CXCR3 plays a critical role in the host defense against cutaneous leishmaniasis caused by *L. major* and helps the Th1 response [35], while CCL17 is associated with diffuse leishmaniasis and late LCL cases [35], [36]. GATA-3 favors the resistance to *L. panamensis* infection, even though promotes the secretion of Th2 cytokines [37]. TNFRSF18 stimulation improves the therapeutic drug response in visceral leishmaniasis [38] and CD23 (FcεRII) participates of the nitric oxide control of *L. braziliensis* infection [39].

We also observed high levels of anti-inflammatory genes in LCL lesions, such as ALOX15, BCL3, LRP1 and SOD3. These genes act by controlling migration and proliferation of inflammatory cells, vascular permeability, response to inflammatory factors, and production of pro-inflammatory cytokines [40]–[43]. We also observed genes involved in the induction of immune tolerance, but none of them had been reported in leishmaniasis.

Controversially, compared with LCL samples, ML samples showed a small number of genes with higher levels of expression related to inflammation and immunity. However, two potentially important genes are ICOS and PPARG. ICOS regulates both Th1 and Th2 responses and is involved in the clearance of *L. mexicana* infection [44]. PPARG was previously associated with susceptibility to leishmaniasis. Activation of PPARγ in macrophages not only induces differentiation of macrophages into the non-inflammatory M2 type but also reduces the resistance to *L. major* infection [45]. Additionally, the presence of increased CXCL13 indicates recruitment of B cells for antibody production [46] and TSLP indicates activation of B lymphocytes, dendritic cells, and Th2 cell differentiation [47]. ABCG2 is a multidrug resistance protein and is involved in the development of Langerhans cells [48].

Taken together, our results using RNA-seq technology and a transcriptomic approach suggest that differences in the quality of the initial immune response of an individual to *L. braziliensis* infection may influence the long term control of the infection. Our observations of the host transcriptome in primary lesions indicate that individuals prone to develop ML may have an insufficient or maladaptive immune response in the cutaneous stage of infection. Such delay in developing specific immunity could be partially responsible for the exacerbated immune response observed in the patients with mucosal leishmaniasis. However, our findings are strictly observational and do not allow us predict the mechanistic underpinnings of this difference. Do ML patients develop a tolerance to *Leishmania* antigens after infection or do they have an intrinsic immunological defect that affects the parasite control?

The detection of a significant number of genes that segregate consistently with the clinical outcomes suggests that transcriptional fingerprints to predict the clinical development of ML may have a functional prognostic significance. Different approaches can be used to answer these questions. We used leave-one-out cross validation analysis to determine the predictive or prognosticating power of the top differently expressed genes. We also used the database offered by IPA software that enumerates genes with previously reported medical importance for prognosis. This literature bias may provide an important reason to study these genes in more detail, but does not directly impact the interpretation of the leave-one-out cross validation results. While we would not expect the resulting lists of genes to overlap (as they did not), they may all have important predictive characteristics when taken together, but used independently. These results encourage us to increase the number of samples to confirm the biological and medical relevance of these results. A larger prospective study to determine positive or negative predictive values and the sensitivity and specificity of these approaches are also needed before clinical use in patients.

In summary, this study provides proof of principle to use next-generation sequencing of the host transcriptome to identify potentially important biological pathways or drug targets in the host-response to *L. braziliensis* infections. Although pathogen transcripts were detected at a low level in the present study, the ability to simultaneously analyze the host and pathogen mRNAs may provide a valuable tool for fundamental pathogenesis research of leishmaniasis or other organisms in the future. Finally, this study suggests that the use of the transcriptional fingerprint or gene expression signature to predict disease outcome is a viable approach. As the costs and time required for next-generation sequencing continue to decrease, this approach may quickly surpass current laboratory tests for both diagnosis and prognosis of many infectious processes.

## Supporting Information

Text S1 **Supporting Materials and Methods.**
(DOC)

Figure S1 **Rarefaction curves were calculated to evaluate the sequencing coverage of the transcriptome in the samples.** Similar numbers of human genes were detected in each sample with no significant difference in the number of genes between the two groups (LCL, n = 5 and ML, n = 5). Only reads uniquely aligned to the human genome were included. LCL: Localized Cutaneous Leishmaniasis. ML: Mucosal Leishmaniasis.
(TIF)

Figure S2 **“Leave-One-Out" permutation analysis of the individual subjects.** Based on groups of 5 ML subjects and 5 LCL subjects and the top 13 differentially expressed genes, the leave-one-out cross validation prediction algorithm correctly predicted the clinical phenotype of 9 out of the 10 samples more accurately than random groupings (*P* = 0.12). The arrow represents the grouping based on the clinical phenotypic classification of 9 out of the 10 subjects correctly.
(TIF)

Table S1 **Reads mapped to host and parasite genomes.** Alignment analysis was performed on each one of the samples. LCL = Localized cutaneous leishmaniasis group. ML = Mucosal leishmaniasis group.
(PDF)

Table S2 **Percentage of multi-hit reads aligned to the human genome.** The analysis was performed on each one of the samples used in this study. LCL = Localized cutaneous leishmaniasis group. ML = Mucosal leishmaniasis group.
(PDF)

Table S3 **Complete list of biological activities upregulated in LCL and ML groups.** The biological activities were determined by Ingenuity Pathway Analysis and are ordered according to P-values range of the LCL group. LCL = Localized cutaneous leishmaniasis group. ML = Mucosal leishmaniasis group.
(PDF)

Table S4 **Biological events related to cancer in LCL and ML samples.** The genes selected by Ingenuity Pathway Analysis and classified under the “cancer biological activity" were re-evaluated by DAVID bioinformatics source and grouped according to gene ontology (GO). The P-values were established by DAVID indicating the importance of the respective GO into the group of genes analyzed. Ratios indicate the proportion of genes observed in the studied samples within the total number of genes that take part in the analyzed event. LCL = Localized cutaneous leishmaniasis group. ML = Mucosal leishmaniasis group.
(PDF)

Table S5 **Biological events related to genetic disorders in LCL and ML samples.** The genes selected by Ingenuity Pathway Analysis and classified within the “genetic disorders" biological activity were re-evaluated by DAVID bioinformatics source and grouped according to gene ontology (GO). The P-values were established by DAVID indicating the importance of the respective GO into the group of genes analyzed. Ratios indicate the proportion of genes observed in the studied samples within the total number of genes that take part in the analyzed event. LCL = Localized cutaneous leishmaniasis group. ML = Mucosal leishmaniasis group.
(PDF)

Table S6 **Biological events related to metabolic diseases in LCL and ML samples.** The genes selected by Ingenuity Pathway Analysis and classified within the “metabolic disease" biological activity were re-evaluated by DAVID bioinformatics source and grouped according to gene ontology (GO). The P-values were established by DAVID indicating the importance of the respective GO into the group of genes analyzed. Ratios indicate the proportion of genes observed in the studied samples within the total number of genes that take part in the analyzed event. LCL = Localized cutaneous leishmaniasis group. ML = Mucosal leishmaniasis group.
(PDF)

Table S7 **Biological events related to cell death in LCL and ML samples.** The genes selected by Ingenuity Pathway Analysis and classified within the “cell death" biological activity were re-evaluated by DAVID bioinformatics source and grouped according to gene ontology (GO). The P-values were established by DAVID indicating the importance of the respective GO into the group of genes analyzed. Ratios indicate the proportion of genes observed within the studied samples in the total number of genes that take part in the analyzed event. LCL = Localized cutaneous leishmaniasis group. ML = Mucosal leishmaniasis group.
(PDF)

Table S8 **Public genome browser websites where the reference genomes used for alignment are available.**
(PDF)

Table S9 **FLAGS used for the quality control during the processing of the reads by SAMtools.**
(PDF)
