# Supplementary material for: Transcriptome Patterns from Primary Cutaneous Leishmania braziliensis Infections Associate with Eventual Development of Mucosal Disease in Humans
Source: PLoS Negl Trop Dis. 2012 Sep 13;6(9):e1816. doi: 10.1371/journal.pntd.0001816 (PMC3441406; doi:10.1371/journal.pntd.0001816)
Supplement: Text S1 — Supporting Materials and Methods. (DOC) [file pntd.0001816.s001.doc]

**Supporting Materials and Methods**

**Transcriptome libraries construction**

***Total RNA isolation:*** The RNA isolation technique used was based on guanidine-chloroform-phenol nucleic acid extraction [1]. Thirty-five 5μm thick sections of frozen 4mm punch skin biopsies were homogenized in 500 μL of denaturing solution for isolation of RNA (Sigma-Aldrich, St. Louis, USA) and the isolation procedure was performed following the manufacturer’s instructions. The total RNA isolated was treated with DNAse (Qiagen, Valencia, USA) and cleaned using RNeasy Micro-kit (Qiagen), all according to manufacturer’s instructions. The RNA concentration of the samples was measured using Nanodrop spectrophotometer and the quality was evaluated using an Agilent Bioanalyzer (Agilent Technologies, Santa Clara, USA).

***cDNA library generation and sequencing:*** The transcriptome libraries were constructed with a mRNA-seq sample preparation kit (Illumina, San Diego, USA) according to the manufacturer’s instructions. mRNA was purified using magnetic beads covered with oligo-dT, fragmented under enzymatic and heat actions, and precipitated with glycogen. The pellet was used as template for double-strand cDNA synthesis. First-strand synthesis was performed using Superscript II kit (Invitrogen) and random primers. The RNA template was subsequently degraded by RNAse H while the second-strand of the cDNA was being synthesized using DNA polymerase I and dNTP. The double strand cDNA was repaired using T4 DNA polymerase and Klenow DNA polymerase. Afterward, a poly-A tail was added at the 3’ end of the DNA and adaptors were bound at both extremities. 200 bp fragments were purified by 2% agarose gel and extracted using QIAquick Gel Extraction kit (Qiagen). Finally, these fragments were amplified by PCR and evaluated by Bioanalyzer. The libraries were sequenced using the Illumina Genome Analyzer II platform.

**Bioinformatic and biostatistic analysis**

***Genome alignment and reads processing:*** The reference genomes where obtained from public genome browser websites (Table S8).

Bowtie aligner (<http://bowtie-bio.sourceforge.net/index.shtml>) and Tophat (<http://tophat.cbcb.umd.edu/> ) were used to aligned the sequenced reads to the reference genomes [2,3]. The parameters used allowed up to 2 mismatches for read alignment to the genome, zero mismatches for read alignment to splice junctions, and inclusion of reads that were assigned to 30 regions or less (multi-hit reads).

SAMtools (<http://samtools.sourceforge.net/> ) was used for quality control and processing of the reads [4]. The quality control step filtered out reads with specific FLAGS (Table S9).

The reads approved by the quality control were then sorted, converted to .BAM files and analyzed by Cufflinks software (<http://cufflinks.cbcb.umd.edu/>).

Cuffdiff from Cufflinks’ package was applied to normalize the number of reads to FPKM (Fragments Per Kilobase per Million) considering both unique and multi-hits reads (up to 30 regions) [5]. Cufflinks will first calculate initial abundance estimates for all transcripts using the uniform dividing scheme. Cufflinks will then re-estimate the abundances dividing each multi-mapped read probabalistically based on the initial abundance estimation of the genes it maps to, the inferred fragment length, and fragment bias. An in-house R script performed the rarefaction curve assay, which was used visualize the transcriptome depth obtained in all samples and only included the reads mapped to unique regions of the human reference genome.

For hierarchical clustering of the samples, we used the software Gene Cluster 3.0 and the Euclidean distance method for both genes and array assays. The linkage was performed in the complete mode (<http://rana.lbl.gov/EisenSoftware.htm>).

***Statistical tests:*** *Student’s t-test* was used to reduce the dimension of the high-throughput data set, considering as threshold *P*-values lower than 0.05. The genes with minimum gene expression fold change of 1.5, both for up and down regulation, were considered for biological interpretation. These thresholds are standards used with this technology and do not imply any individual gene is statistically significant, but rather allow the inclusion of 5% of false positive genes and genes with at least 50% of increase in the expression, respectively. Linear regression analysis was applied to verify the similarity of gene expression between LCL and ML samples. MA-plot was used to display the gene expression distribution in both groups.

An in-house R script was used to perform the leave-one-out cross-validation. The program generated all possible groupings among the samples by permuting the phenotype labels of the samples. For each permutation, the program left one sample out and used the rest samples to predict its phenotype based on the expression level of the top differentially expressed genes. The classification was performed based on the gene voting algorithm of Golub et al. [6], with minor modifications as described in Bleharski et al. [7]. P value was calculated based on the distribution of prediction accuracy of the grouping that was assigned correctly among all possible groupings.

Supporting References

1. Chomczynski P, Sacchi N (1987) Single-step method of RNA isolation by acid guanidinium thiocyanate-phenol-chloroform extraction. Anal Biochem 162: 156-159.

2. Langmead B, Trapnell C, Pop M, Salzberg SL (2009) Ultrafast and memory-efficient alignment of short DNA sequences to the human genome. Genome Biol 10: R25.

3. Trapnell C, Pachter L, Salzberg SL (2009) TopHat: discovering splice junctions with RNA-Seq. Bioinformatics 25: 1105-1111.

4. Li H, Handsaker B, Wysoker A, Fennell T, Ruan J, Homer N, Marth G, Abecasis G, Durbin R (2009) The Sequence Alignment/Map format and SAMtools. Bioinformatics 25: 2078-2079.

5. Trapnell C, Williams BA, Pertea G, Mortazavi A, Kwan G, van Baren MJ, Salzberg SL, Wold BJ, Pachter L (2010) Transcript assembly and quantification by RNA-Seq reveals unannotated transcripts and isoform switching during cell differentiation. Nat Biotechnol 28: 511-515.

6. Golub TR, Slonim DK, Tamayo P, Huard C, Gaasenbeek M, Mesirov JP, Coller H, Loh ML, Downing JR, Caligiuri MA, Bloomfield CD, Lander ES (1999) Molecular classification of cancer: class discovery and class prediction by gene expression monitoring. Science 286: 531-537.

7. Bleharski JR, Li H, Meinken C, Graeber TG, Ochoa MT, Yamamura M, Burdick A, Sarno EN, Wagner M, Rollinghoff M, Rea TH, Colonna M, Stenger S, Bloom BR, Eisenberg D, Modlin RL (2003) Use of genetic profiling in leprosy to discriminate clinical forms of the disease. Science 301: 1527-1530.
