# Supplementary figures and images for: Transcriptome Patterns from Primary Cutaneous Leishmania braziliensis Infections Associate with Eventual Development of Mucosal Disease in Humans
Source: PLoS Negl Trop Dis. 2012 Sep 13;6(9):e1816. doi: 10.1371/journal.pntd.0001816 (PMC3441406; doi:10.1371/journal.pntd.0001816)

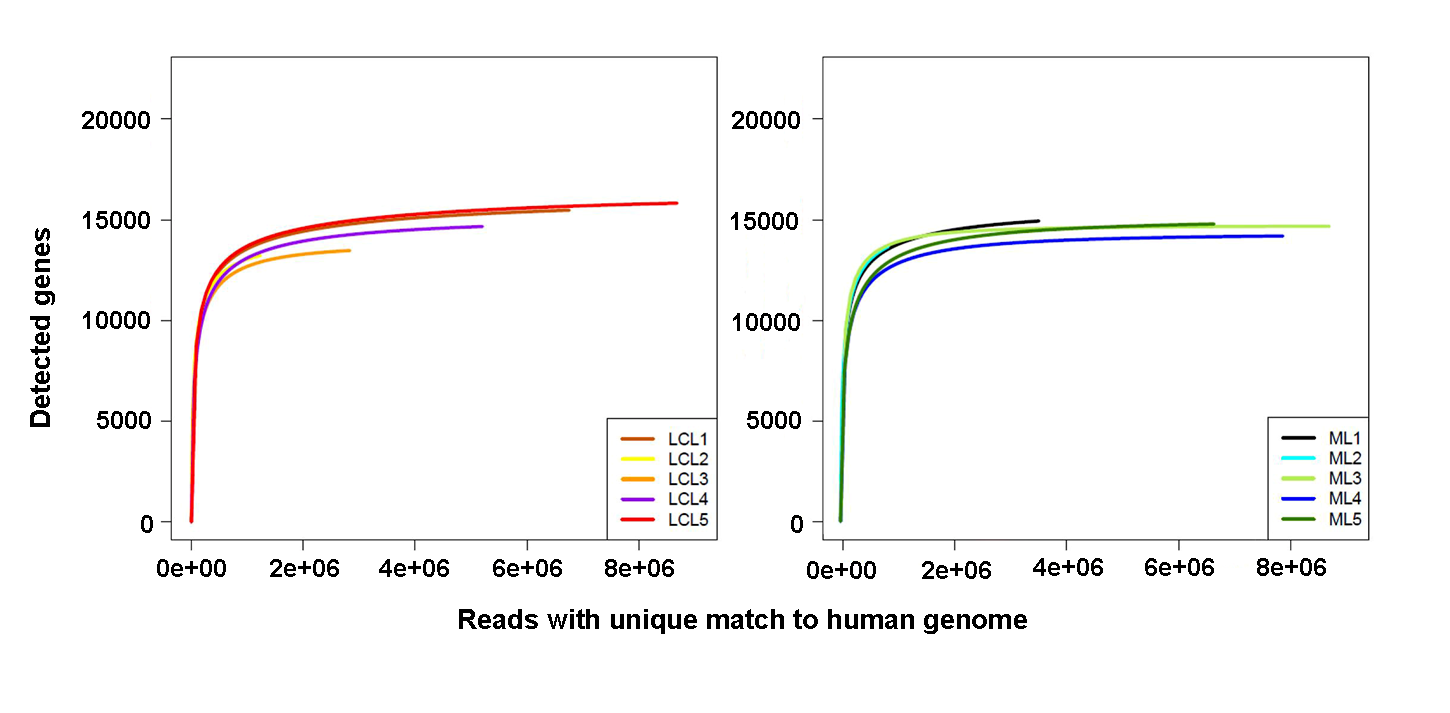

Supplement: Figure S1 — Rarefaction curves were calculated to evaluate the sequencing coverage of the transcriptome in the samples. Similar numbers of human genes were detected in each sample with no significant difference in the number of genes between the two groups (LCL, n = 5 and ML, n = 5). Only reads uniquely aligned to the human genome were included. LCL: Localized Cutaneous Leishmaniasis. ML: Mucosal Leishmaniasis. (TIF) [file pntd.0001816.s002.tif]

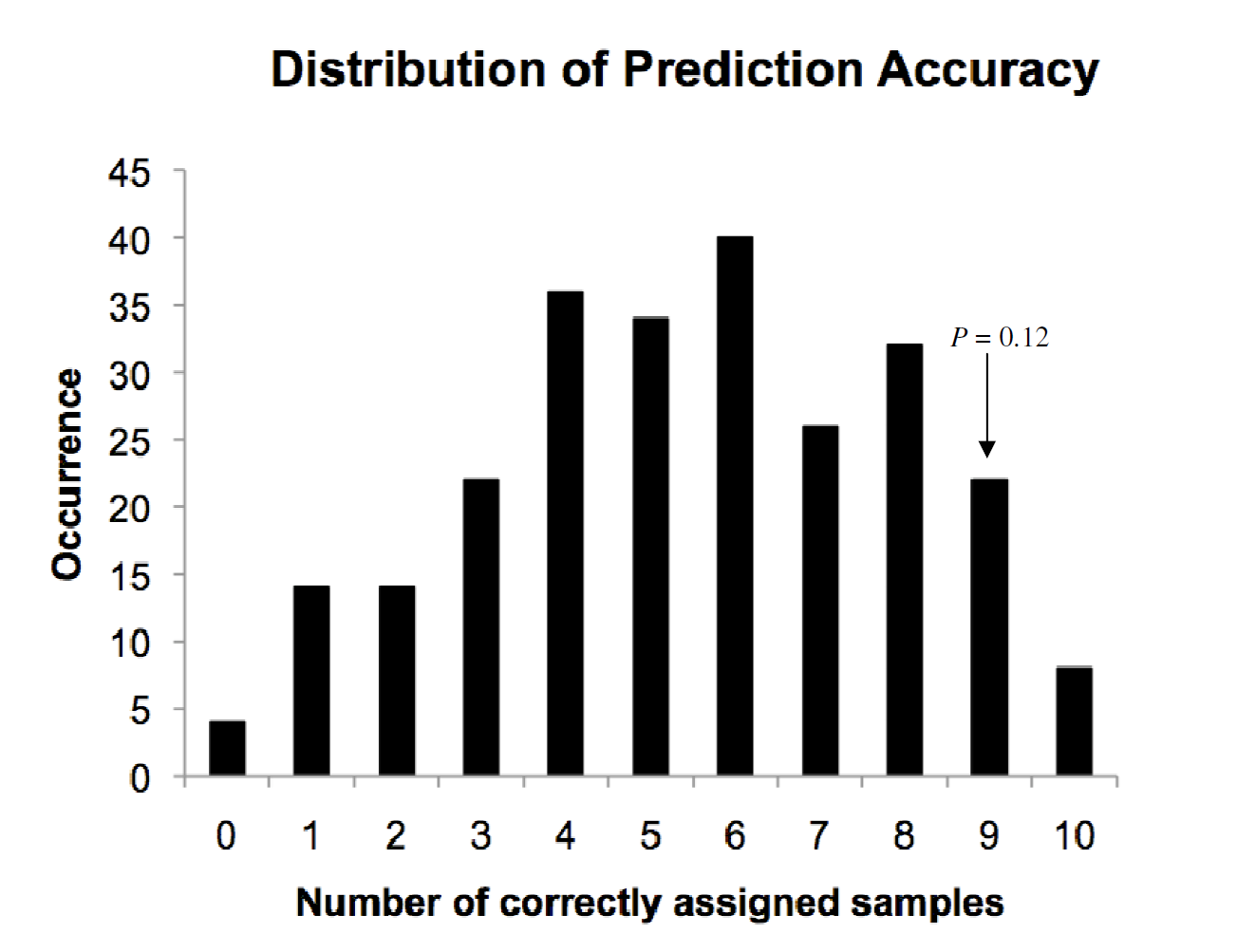

Supplement: Figure S2 — “Leave-One-Out" permutation analysis of the individual subjects. Based on groups of 5 ML subjects and 5 LCL subjects and the top 13 differentially expressed genes, the leave-one-out cross validation prediction algorithm correctly predicted the clinical phenotype of 9 out of the 10 samples more accurately than random groupings (P = 0.12). The arrow represents the grouping based on the clinical phenotypic classification of 9 out of the 10 subjects correctly. (TIF) [file pntd.0001816.s003.tif]
