# Supplementary material for: Transcriptome Patterns from Primary Cutaneous Leishmania braziliensis Infections Associate with Eventual Development of Mucosal Disease in Humans
Source: PLoS Negl Trop Dis. 2012 Sep 13;6(9):e1816. doi: 10.1371/journal.pntd.0001816 (PMC3441406; doi:10.1371/journal.pntd.0001816)
Supplement: Table S1 — Reads mapped to host and parasite genomes. Alignment analysis was performed on each one of the samples. LCL = Localized cutaneous leishmaniasis group. ML = Mucosal leishmaniasis group. (PDF) [file pntd.0001816.s004.pdf]

Table S1.

Reads mapped to host and parasite genomes.

Samples	Reads aligned to human genome (%)	Reads aligned to *L. braziliensis* genome (%)	Reads aligned to both genomes (%)	Unaligned reads (%)
LCL-01	79.42	0.07	0.002	20.50
LCL-02	78.03	0.25	0.004	21.72
LCL-03	80.23	0.18	0.003	19.59
LCL-04	82.63	0.07	0.002	17.29
LCL-05	77.23	0.17	0.003	22.59
**Average**	**79.51**	**0.15**	**0.003**	**20.34**
**Median**	**79.42**	**0.17**	**0.003**	**20.50**
ML-01	64.87	0.79	0.008	34.34
ML-02	59.13	0.91	0.010	39.96
ML-03	82.38	0.06	0.002	17.56
ML-04	80.14	0.06	0.001	19.80
ML-05	80.79	0.09	0.002	19.13
**Average**	**73.46**	**0.38**	**0.005**	**26.16**
**Median**	**80.14**	**0.09**	**0.002**	**19.80**

Alignment analysis was performed on each one of the samples. LCL = Localized cutaneous leishmaniasis group. ML = Mucosal leishmaniasis group.
