# Supplementary material for: Transcriptome Patterns from Primary Cutaneous Leishmania braziliensis Infections Associate with Eventual Development of Mucosal Disease in Humans
Source: PLoS Negl Trop Dis. 2012 Sep 13;6(9):e1816. doi: 10.1371/journal.pntd.0001816 (PMC3441406; doi:10.1371/journal.pntd.0001816)
Supplement: Table S2 — Percentage of multi-hit reads aligned to the human genome. The analysis was performed on each one of the samples used in this study. LCL = Localized cutaneous leishmaniasis group. ML = Mucosal leishmaniasis group. (PDF) [file pntd.0001816.s005.pdf]

Table S2.

Percentage of multi-hit reads aligned to the human genome.

Samples	Reads matched to 1 unique region (%)	Reads matched to 2 to 30 regions (%)	Reads matched to more than 30 regions (%)
LCL-01	82.68	17.10	0.21
LCL-02	81.93	17.60	0.48
LCL-03	78.74	20.82	0.44
LCL-04	75.94	23.65	0.41
LCL-05	77.45	22.28	0.27
**Average**	**79.35**	**20.29**	**0.36**
**Median**	**78.74**	**20.82**	**0.41**
ML-01	76.09	22.97	0.93
ML-02	73.32	25.71	0.97
ML-03	79.88	19.54	0.58
ML-04	71.01	28.80	0.19
ML-05	69.62	30.12	0.26
**Average**	**73.98**	**25.43**	**0.59**
**Median**	**73.32**	**25.71**	**0.58**

The analysis was performed on each one of the samples used in this study.

LCL = Localized cutaneous leishmaniasis group. ML = Mucosal leishmaniasis group.
