# Supplementary material for: Transcriptome Patterns from Primary Cutaneous Leishmania braziliensis Infections Associate with Eventual Development of Mucosal Disease in Humans
Source: PLoS Negl Trop Dis. 2012 Sep 13;6(9):e1816. doi: 10.1371/journal.pntd.0001816 (PMC3441406; doi:10.1371/journal.pntd.0001816)
Supplement: Table S3 — Complete list of biological activities upregulated in LCL and ML groups. The biological activities were determined by Ingenuity Pathway Analysis and are ordered according to P-values range of the LCL group. LCL = Localized cutaneous leishmaniasis group. ML = Mucosal leishmaniasis group. (PDF) [file pntd.0001816.s006.pdf]

Table S3.

Complete list of biological activities upregulated in LCL and ML groups.

	LCL		ML	
**Biological Functions**	***P*-values**	**# Genes**	***P*-values**	**# Genes**
Cell-To-Cell Signaling and Interaction	8.49E-09 - 1.85E-02	75	1.15E-02 - 4.95E-02	9
Immune Cell Trafficking	8.75E-08 - 1.85E-02	47	4.02E-03 - 4.95E-02	3
Inflammatory Response	8.75E-08 - 1.92E-02	59	1.68E-02 - 3.33E-02	2
Infectious Disease	1.07E-06 - 1.85E-02	62	3.33E-02 - 4.88E-02	3
Cellular Growth and Proliferation	1.09E-06 - 1.85E-02	89	1.68E-02 - 4.54E-02	27
Dermatological Diseases and Conditions	8.35E-06 - 1.99E-02	62	1.68E-02 - 1.68E-02	5
Immunological Disease	1.95E-05 - 1.85E-02	80	1.17E-02 - 4.95E-02	34
Cellular Function and Maintenance	2.69E-05 - 1.85E-02	39	4.30E-03 - 3.33E-02	21
Inflammatory Disease	7.93E-05 - 1.99E-02	95	8.64E-04 - 4.88E-02	12
Cancer	1.63E-04 - 1.85E-02	101	2.80E-04 - 4.95E-02	80
Small Molecule Biochemistry	2.08E-04 - 1.85E-02	21	9.33E-03 - 3.97E-02	17
Genetic Disorder	3.10E-04 - 1.85E-02	177	2.80E-04 - 4.88E-02	164
Metabolic Disease	3.27E-04 - 1.85E-02	14	2.80E-04 - 4.95E-02	73
Hair and Skin Development and Function	3.41E-04 - 1.85E-02	16	1.15E-02 - 4.37E-02	13
Cell-mediated Immune Response	3.51E-04 - 1.85E-02	27	4.02E-03 - 4.95E-02	2
Cell Morphology	4.85E-04 - 1.98E-02	40	2.98E-03 - 3.52E-02	30
Cellular Movement	5.83E-04 - 1.85E-02	56	4.02E-03 - 4.95E-02	16
Tissue Development	8.37E-04 - 1.92E-02	59	3.05E-03 - 4.95E-02	19
Gene Expression	1.01E-03 - 1.85E-02	52	2.71E-03 - 4.95E-02	10
Tissue Morphology	1.16E-03 - 1.92E-02	40	9.72E-03 - 4.36E-02	17
Antigen Presentation	1.38E-03 - 1.85E-02	18	1.68E-02 - 1.68E-02	1
Infection Mechanism	1.07E-03 - 1.85E-02	41	1.68E-02 - 1.68E-02	1
Connective Tissue Development and Function	1.89E-03 - 1.85E-02	7	3.05E-03 - 4.95E-02	18
Cellular Assembly and Organization	2.42E-03 - 1.86E-02	22	2.22E-03 - 4.95E-02	23
Cell Death	3.29E-03 - 1.85E-02	77	8.31E-04 - 4.86E-02	37
Cell Cycle	4.87E-03 - 1.85E-02	8	2.79E-03 - 4.77E-02	37
Cell Signaling	1.13E-02 - 1.85E-02	6	9.33E-03 - 3.97E-02	4
Cellular Compromise	1.85E-02 - 1.85E-02	6	5.56E-03 - 3.33E-02	13
DNA Replication, Recombination, and Repair	1.85E-02 - 1.85E-02	1	1.68E-02 - 3.33E-02	5
Humoral Immune Response	1.85E-02 - 1.85E-02	1	1.68E-02 - 3.33E-02	3
Nucleic Acid Metabolism	1.85E-02 - 1.85E-02	1	2.98E-03 - 3.97E-02	9
Post-Translational Modification	1.85E-02 - 1.85E-02	3	1.97E-02 - 3.33E-02	14
Vitamin and Mineral Metabolism	1.85E-02 - 1.85E-02	1	1.15E-02 - 3.33E-02	5

The biological activities were determined by Ingenuity Pathway Analysis and are ordered according to P-values range of the LCL group.

LCL = Localized cutaneous leishmaniasis group. ML = Mucosal leishmaniasis group.
