# Supplementary material for: Transcriptome Patterns from Primary Cutaneous Leishmania braziliensis Infections Associate with Eventual Development of Mucosal Disease in Humans
Source: PLoS Negl Trop Dis. 2012 Sep 13;6(9):e1816. doi: 10.1371/journal.pntd.0001816 (PMC3441406; doi:10.1371/journal.pntd.0001816)
Supplement: Table S4 — Biological events related to cancer in LCL and ML samples. The genes selected by Ingenuity Pathway Analysis and classified under the “cancer biological activity" were re-evaluated by DAVID bioinformatics source and grouped according to gene ontology (GO). The P-values were established by DAVID indicating the importance of the respective GO into the group of genes analyzed. Ratios indicate the proportion of genes observed in the studied samples within the total number of genes that take part in the analyzed event. LCL = Localized cutaneous leishmaniasis group. ML = Mucosal leishmaniasis group. (PDF) [file pntd.0001816.s007.pdf]

Table S4.

Biological events related to cancer in LCL and ML samples.

**LCL**		
**Events**	***P*-value**	**Ratio**
GO:0006952~defense response	6.07E-04	13/615 (0.021)
GO:0032680~regulation of tumor necrosis factor production	0.001048	4/31(0.129)
GO:0045582~positive regulation of T cell differentiation	0.001151	4/32 (0.125)
GO:0045621~positive regulation of lymphocyte differentiation	0.001498	4/35 (0.114)
GO:0006955~immune response	0.001644	13/690 (0.019)
GO:0046649~lymphocyte activation	0.001873	7/199 (0.035)
GO:0008283~cell proliferation	0.002076	10/436 (0.023)
GO:0043066~negative regulation of apoptosis	0.002115	9/354 (0.025)
GO:0060548~negative regulation of cell death	0.002347	9/360 (0.025)
GO:0001775~cell activation	0.002638	8/287 (0.028)
GO:0045580~regulation of T cell differentiation	0.004422	4/51 (0.078)
GO:0045321~leukocyte activation	0.004922	7/242 (0.029)
GO:0016064~immunoglobulin mediated immune response	0.005194	4/54 (0.074)
GO:0006954~inflammatory response	0.005214	8/325 (0.025)
GO:0042981~regulation of apoptosis	0.005703	13/804 (0.016)
GO:0019724~B cell mediated immunity	0.00575	4/56 (0.071)
GO:0043067~regulation of programmed cell death	0.006161	13/812 (0.016)
GO:0010941~regulation of cell death	0.00634	13/815 (0.016)
GO:0009411~response to UV	0.00665	4/59 (0.068)
GO:0009611~response to wounding	0.007443	10/530 (0.019)
GO:0045619~regulation of lymphocyte differentiation	0.007974	4/63 (0.063)
**ML**		
**Events**	***P*-value**	**Ratio**
GO:0042127~regulation of cell proliferation	3.72E-04	13/787 (0.017)
GO:0007346~regulation of mitotic cell cycle	8.63E-04	6/152 (0.039)
GO:0008285~negative regulation of cell proliferation	0.001831	8/361 (0.022)
GO:0007584~response to nutrient	0.004822	5/140 (0.036)
GO:0050678~regulation of epithelial cell proliferation	0.005004	4/71 (0.056)
GO:0051726~regulation of cell cycle	0.005431	7/331 (0.021)
GO:0050680~negative regulation of epithelial cell proliferation	0.006542	3/25 (0.120)
GO:0010605~negative regulation of macromolecule metabolic process	0.008959	10/734 (0.014)
GO:0003006~reproductive developmental process	0.008969	6/262 (0.023)
GO:0051338~regulation of transferase activity	0.009423	7/372 (0.019)

The genes selected by Ingenuity Pathway Analysis and classified under the "cancer biological activity" were re-evaluated by DAVID bioinformatics source and grouped according to gene ontology (GO). The P-values were established by DAVID indicating the importance of the respective GO into the group of genes analyzed. Ratios indicate the proportion of genes observed in the studied samples within the total number of genes that take part in the analyzed event. LCL = Localized cutaneous leishmaniasis group. ML = Mucosal leishmaniasis group.
