# Supplementary material for: Transcriptome Patterns from Primary Cutaneous Leishmania braziliensis Infections Associate with Eventual Development of Mucosal Disease in Humans
Source: PLoS Negl Trop Dis. 2012 Sep 13;6(9):e1816. doi: 10.1371/journal.pntd.0001816 (PMC3441406; doi:10.1371/journal.pntd.0001816)
Supplement: Table S5 — Biological events related to genetic disorders in LCL and ML samples. The genes selected by Ingenuity Pathway Analysis and classified within the “genetic disorders" biological activity were re-evaluated by DAVID bioinformatics source and grouped according to gene ontology (GO). The P-values were established by DAVID indicating the importance of the respective GO into the group of genes analyzed. Ratios indicate the proportion of genes observed in the studied samples within the total number of genes that take part in the analyzed event. LCL = Localized cutaneous leishmaniasis group. ML = Mucosal leishmaniasis group. (PDF) [file pntd.0001816.s008.pdf]

Table S5.

Biological events related to genetic disorders in LCL and ML samples.

**LCL**		
**Events**	***P*-value**	**Ratios**
GO:0006955~immune response	1.09E-04	21/690 (0.030)
GO:0032680~regulation of tumor necrosis factor production	4.00E-04	5/31 (0.161)
GO:0003013~circulatory system process	0.001301	9/186 (0.048)
GO:0002684~positive regulation of immune system process	0.001613	10/238 (0.042)
GO:0019882~antigen processing and presentation	0.002549	6/83 (0.072)
GO:0044057~regulation of system process	0.002834	11/309 (0.036)
GO:0016064~immunoglobulin mediated immune response	0.003283	5/54 (0.093)
GO:0019724~B cell mediated immunity	0.003747	5/56 (0.089)
GO:0032319~regulation of Rho GTPase activity	0.003846	4/28 (0.143)
GO:0001775~cell activation	0.005589	10/287 (0.035)
GO:0002504~antigen processing and presentation of peptide or polysaccharide antigen via MHC class II	0.006146	4/33 (0.121)
GO:0045581~negative regulation of T cell differentiation	0.00662	3/11 (0.273)
GO:0009611~response to wounding	0.007533	14/530 (0.026)
GO:0046649~lymphocyte activation	0.007745	8/199 (0.040)
GO:0002920~regulation of humoral immune response	0.007885	3/12 (0.250)
GO:0002449~lymphocyte mediated immunity	0.008277	5/70 (0.071)
**ML**		
**Events**	***P*-value**	**Ratios**
GO:0043065~positive regulation of apoptosis	0.003853	11/430 (0.026)
GO:0043068~positive regulation of programmed cell death	0.004046	11/433 (0.025)
GO:0010941~regulation of cell death	0.00407	16/815 (0.020)
GO:0010942~positive regulation of cell death	0.004179	11/435 (0.025)
GO:0001942~hair follicle development	0.005529	4/42 (0.095)
GO:0008219~cell death	0.008572	14/719 (0.019)
GO:0042981~regulation of apoptosis	0.008815	15/804 (0.019)
GO:0043067~regulation of programmed cell death	0.009571	15/812 (0.018)
GO:0007346~regulation of mitotic cell cycle	0.009916	6/152 (0.039)

The genes selected by Ingenuity Pathway Analysis and classified within the "genetic disorders" biological activity were re-evaluated by DAVID bioinformatics source and grouped according to gene ontology (GO). The P-values were established by DAVID indicating the importance of the respective GO into the group of genes analyzed. Ratios indicate the proportion of genes observed in the studied samples within the total number of genes that take part in the analyzed event.

LCL = Localized cutaneous leishmaniasis group. ML = Mucosal leishmaniasis group.
