# Supplementary material for: Transcriptome Patterns from Primary Cutaneous Leishmania braziliensis Infections Associate with Eventual Development of Mucosal Disease in Humans
Source: PLoS Negl Trop Dis. 2012 Sep 13;6(9):e1816. doi: 10.1371/journal.pntd.0001816 (PMC3441406; doi:10.1371/journal.pntd.0001816)
Supplement: Table S6 — Biological events related to metabolic diseases in LCL and ML samples. The genes selected by Ingenuity Pathway Analysis and classified within the “metabolic disease" biological activity were re-evaluated by DAVID bioinformatics source and grouped according to gene ontology (GO). The P-values were established by DAVID indicating the importance of the respective GO into the group of genes analyzed. Ratios indicate the proportion of genes observed in the studied samples within the total number of genes that take part in the analyzed event. LCL = Localized cutaneous leishmaniasis group. ML = Mucosal leishmaniasis group. (PDF) [file pntd.0001816.s009.pdf]

Table S6.

Biological events related to metabolic diseases in LCL and ML samples.

**LCL**		
**Events**	***P*-value**	**Ratio**
GO:0006952~defense response	1.81E-04	6/615 (0.010)
GO:0016064~immunoglobulin mediated immune response	0.001186	3/54 (0.056)
GO:0019724~B cell mediated immunity	0.001275	3/56 (0.054)
GO:0002449~lymphocyte mediated immunity	0.001984	3/70 (0.043)
GO:0002250~adaptive immune response	0.002395	3/77 (0.039)
GO:0002460~adaptive immune response based on somatic recombination of immune receptors built from immunoglobulin superfamily domains	0.002395	3/77 (0.039)
GO:0051605~protein maturation by peptide bond cleavage	0.002977	3/86 (0.035)
GO:0002443~leukocyte mediated immunity	0.002977	3/86 (0.035)
GO:0006955~immune response	0.003314	5/690 (0.007)
GO:0016485~protein processing	0.004992	3/112 (0.027)
GO:0051604~protein maturation	0.005896	3/122 (0.025)
GO:0002252~immune effector process	0.007072	3/134 (0.022)
**ML**		
**Events**	***P*-value**	**Ratio**
GO:0015677~copper ion import	0.007526	2/2 (1.000)

The genes selected by Ingenuity Pathway Analysis and classified within the "metabolic disease" biological activity were re-evaluated by DAVID bioinformatics source and grouped according to gene ontology (GO). The P-values were established by DAVID indicating the importance of the respective GO into the group of genes analyzed. Ratios indicate the proportion of genes observed in the studied samples within the total number of genes that take part in the analyzed event.

LCL = Localized cutaneous leishmaniasis group. ML = Mucosal leishmaniasis group.
