# Supplementary material for: Transcriptome Patterns from Primary Cutaneous Leishmania braziliensis Infections Associate with Eventual Development of Mucosal Disease in Humans
Source: PLoS Negl Trop Dis. 2012 Sep 13;6(9):e1816. doi: 10.1371/journal.pntd.0001816 (PMC3441406; doi:10.1371/journal.pntd.0001816)
Supplement: Table S7 — Biological events related to cell death in LCL and ML samples. The genes selected by Ingenuity Pathway Analysis and classified within the “cell death" biological activity were re-evaluated by DAVID bioinformatics source and grouped according to gene ontology (GO). The P-values were established by DAVID indicating the importance of the respective GO into the group of genes analyzed. Ratios indicate the proportion of genes observed within the studied samples in the total number of genes that take part in the analyzed event. LCL = Localized cutaneous leishmaniasis group. ML = Mucosal leishmaniasis group. (PDF) [file pntd.0001816.s010.pdf]

**Table S7.**

**Biological events related to cell death in LCL and ML samples.**

| **LCL** | | |
|---|---|---|
| **Events** | ***P*-value** | **Ratio** |
| GO:0043067~regulation of programmed cell death | 8.00E-08 | 19/812 (0.023) |
| GO:0032680~regulation of tumor necrosis factor production | 1.76E-05 | 5/31 (0.161) |
| GO:0046649~lymphocyte activation | 6.69E-05 | 8/199 (0.040) |
| GO:0043069~negative regulation of programmed cell death | 8.21E-05 | 10/359 (0.028) |
| GO:0045321~leukocyte activation | 2.26E-04 | 8/242 (0.033) |
| GO:0006952~defense response | 2.58E-04 | 12/615 (0.020) |
| GO:0042110~T cell activation | 4.52E-04 | 6/126 (0.048) |
| GO:0007242~intracellular signaling cascade | 4.65E-04 | 17/1256 (0.014) |
| GO:0006916~anti-apoptosis | 6.26E-04 | 7/206 (0.034) |
| GO:0001775~cell activation | 6.30E-04 | 8/287 (0.028) |
| GO:0008283~cell proliferation | 0.001632 | 9/436 (0.021) |
| GO:0001817~regulation of cytokine production | 0.002283 | 6/181 (0.033) |
| GO:0002521~leukocyte differentiation | 0.004467 | 5/131 (0.038) |
| GO:0043085~positive regulation of catalytic activity | 0.004813 | 9/520 (0.017) |
| GO:0045860~positive regulation of protein kinase activity | 0.00556 | 6/223 (0.027) |
| GO:0010557~positive regulation of macromolecule biosynthetic process | 0.005776 | 10/654 (0.015) |
| GO:0033674~positive regulation of kinase activity | 0.006438 | 6/231 (0.026) |
| GO:0031343~positive regulation of cell killing | 0.006582 | 3/24 (0.125) |
| GO:0030097~hemopoiesis | 0.007034 | 6/236 (0.025) |
| GO:0051347~positive regulation of transferase activity | 0.007537 | 6/240 (0.025) |
| GO:0006955~immune response | 0.008114 | 10/690 (0.014) |
| GO:0045859~regulation of protein kinase activity | 0.008194 | 7/345 (0.020) |
| GO:0009891~positive regulation of biosynthetic process | 0.008488 | 10/695 (0.014) |
| GO:0031341~regulation of cell killing | 0.008897 | 3/28 (0.107) |
| GO:0043549~regulation of kinase activity | 0.009608 | 7/357 (0.020) |
| GO:0044093~positive regulation of molecular function | 0.009659 | 9/586 (0.015) |
| **ML** | | |
| **Events** | ***P*-value** | **Ratio** |
| GO:0043067~regulation of programmed cell death | 3.41E-08 | 14/812 (0.017) |
| GO:0043068~positive regulation of programmed cell death | 6.70E-08 | 11/433 (0.025) |
| GO:0042127~regulation of cell proliferation | 1.02E-04 | 10/787 (0.013) |
| GO:0012502~induction of programmed cell death | 1.31E-04 | 7/321 (0.022) |
| GO:0006915~apoptosis | 6.28E-04 | 8/602 (0.013) |
| GO:0007584~response to nutrient | 0.005134 | 4/140 (0.029) |
| GO:0007346~regulation of mitotic cell cycle | 0.006448 | 4/152 (0.026) |
| GO:0010629~negative regulation of gene expression | 0.008013 | 6/504 (0.012) |
| GO:0010033~response to organic substance | 0.008397 | 7/721 (0.010) |
| GO:0010605~negative regulation of macromolecule metabolic process | 0.009135 | 7/734 (0.010) |
| GO:0051726~regulation of cell cycle | 0.00915 | 5/331 (0.015) |

The genes selected by Ingenuity Pathway Analysis and classified within the "cell death" biological activity were re-evaluated by DAVID bioinformatics source and grouped according to gene ontology (GO). The P-values were established by DAVID indicating the importance of the respective GO into the group of genes analyzed. Ratios indicate the proportion of genes observed within the studied samples in the total number of genes that take part in the analyzed event. LCL = Localized cutaneous leishmaniasis group. ML = Mucosal leishmaniasis group.
