# Supplementary material for: Transcriptome Patterns from Primary Cutaneous Leishmania braziliensis Infections Associate with Eventual Development of Mucosal Disease in Humans
Source: PLoS Negl Trop Dis. 2012 Sep 13;6(9):e1816. doi: 10.1371/journal.pntd.0001816 (PMC3441406; doi:10.1371/journal.pntd.0001816)
Supplement: Table S8 — Public genome browser websites where the reference genomes used for alignment are available. (PDF) [file pntd.0001816.s011.pdf]

Table S8.

Public genome browser websites where the reference genomes used for alignment are available.

Organism	Websites	Access ID
Homo sapiens	USCS Genome Browser<br>http://genome.ucsc.edu/	Feb. 2009<br>(hg19, GRCh37)
Leishmania braziliensis	TritrypDB<br>http://tritrypdb.org/tritrypdb/	Release 3.2
Staphylococcus aureus	Wellcome Trust Sanger Institute<br>http://www.sanger.ac.uk/	EMRSA15
Pseudomonas aeruginosa	Wellcome Trust Sanger Institute<br>http://www.sanger.ac.uk/	LEB58
