# Supplementary material for: Transcriptome Patterns from Primary Cutaneous Leishmania braziliensis Infections Associate with Eventual Development of Mucosal Disease in Humans
Source: PLoS Negl Trop Dis. 2012 Sep 13;6(9):e1816. doi: 10.1371/journal.pntd.0001816 (PMC3441406; doi:10.1371/journal.pntd.0001816)
Supplement: Table S9 — FLAGS used for the quality control during the processing of the reads by SAMtools. (PDF) [file pntd.0001816.s012.pdf]

Table S9.

FLAGS used for the quality control during the processing of the reads by SAMtools.

Flags	Description
0x4	the query sequence itself is unmapped
0x100	the alignment is not primary
0x200	the read fails platform/vendor quality checks
0x400	the read is either a PCR or an optical duplicate
